# Clinical Score to Predict Recurrence in Patients with Stage II and Stage III Colon Cancer

**DOI:** 10.3390/cancers14235891

**Published:** 2022-11-29

**Authors:** David Viñal, Sergio Martinez-Recio, Daniel Martinez-Perez, Iciar Ruiz-Gutierrez, Diego Jimenez-Bou, Jesús Peña-Lopez, Maria Alameda-Guijarro, Gema Martin-Montalvo, Antonio Rueda-Lara, Laura Gutierrez-Sainz, Maria Elena Palacios, Ana Belén Custodio, Ismael Ghanem, Jaime Feliu, Nuria Rodríguez-Salas

**Affiliations:** 1 Department of Medical Oncology, Hospital Universitario La Paz, 28046 Madrid, Spain; 2 Department of Pathology, Hospital Universitario La Paz, 28046 Madrid, Spain; 3 Department of Medical Oncology, Hospital Universitario La Paz, IdiPAZ, Catedra UAM-AMGEN, CIBERONC, 28046 Madrid, Spain; 4 Department of Medical Oncology, Hospital Universitario La Paz, IdiPAZ, CIBERONC, 28046 Madrid, Spain

**Keywords:** colonic neoplasms, chemotherapy, adjuvant, tumor budding

## Abstract

**Simple Summary:**

The prognosis of patients with stage II and stage III colon cancer is heterogeneous. Clinical and pathological characteristics may help to further refine the recurrence risk. We built a prognostic score and categorized patients into two risk groups in a training and validation cohort. We assigned two points to T4 and one point to N2 and high tumor budding based on the multivariate cox regression analysis for time to recurrence (TTR) in the training cohort. Forty-five percent of the patients were assigned to the low-risk group and compared to the high-risk group, had a significantly longer TTR. These results were confirmed in the validation cohort.

**Abstract:**

Background: The prognosis of patients with stage II and stage III colon cancer is heterogeneous. Clinical and pathological characteristics, such as tumor budding, may help to further refine the recurrence risk. Methods: We included all the patients with localized colon cancer at Hospital Universitario La Paz from October 2016 to October 2021. We built a prognostic score for recurrence in the training cohort based on multivariate cox regression analysis and categorized the patients into two risk groups. Results: A total of 440 patients were included in the training cohort. After a median follow-up of 45 months, 81 (18%) patients had a first tumor recurrence. T4, N2, and high tumor budding remained with a *p* value <0.05 at the last step of the multivariate cox regression model for time to recurrence (TTR). We assigned 2 points to T4 and 1 point to N2 and high tumor budding. Forty-five percent of the patients were assigned to the low-risk group (score = 0). Compared to the high-risk group (score 1–4), patients in the low-risk group had a significantly longer TTR (hazard ratio for disease recurrence of 0.14 (95%CI: 0.00 to 0.90; *p* < 0.045)). The results were confirmed in the validation cohort. Conclusions: In our study, we built a simple score to predict tumor recurrence based on T4, N2, and high tumor budding. Patients in the low-risk group, that comprised 44% of the cohort, had an excellent prognosis.

## 1. Introduction

Colorectal cancer is the third most common tumor and the second cause of cancer-related cause of death globally [1]. In patients with stage II and stage III colon cancer, the prognosis is heterogeneous, and survival varies depending on numerous factors. Classically, for the pathologic stage at diagnosis, according to the American Joint Committee on Cancer (AJCC)/Union for International Cancer Control (UICC), the tumor, node, and metastasis (TNM) staging classification was considered the most important indicator of outcome [2]. However, patients with stage IIIa disease may have a more favorable prognosis than patients with IIb stage, which may indicate that other factors contribute significantly to the prognosis of the patient. Globally, it is estimated that 35% of the patients will eventually recur [3]. To further improve the outcome, chemotherapy has been established as a standard of care for stage III colon cancer with a 10 to 20% of survival benefit (depending on the regimen of chemotherapy) and is an option for patients with intermediate- or high-risk stage II [4,5]. The latest European Society for Medical Oncology (ESMO) guidelines include lymph node sampling <12 and T4 stage including perforation as major prognostic factors and high-grade tumor, vascular invasion, lymphatic invasion, perineural invasion, tumor presentation with obstruction, and high preoperative carcinoembryonic antigen (CEA) levels as minor prognostic factors. The National Comprehensive Cancer Network (NCCN) also includes high tumor budding and close, indeterminate, or positive margins as risk factors for recurrence. To better define the prognosis and recurrence risk of patients with resected colon cancer, several nomograms have been published [6,7,8]. One of the most widely used is the Memorial Sloan Kettering Cancer Center (MSKCC) colon cancer recurrence nomogram, which predicts freedom from recurrence based on nine clinicopathological features including age, tumor size, preoperative carcinoembryonic antigen (CEA), use of adjuvant chemotherapy, and other indicators of tumor invasiveness [6]. A recently published update simplified the score to five items; however, tumor-infiltrating lymphocytes were included in the nomogram, a feature not available in many centers [8].

The aim of this study is to create a simple clinical score to predict recurrence using clinical and pathological variables available in routine clinical practice and to select a subgroup of patients with excellent prognosis according to this score.

## 2. Materials and Methods

This is a single-institution retrospective observational study. We included all patients who underwent curative surgery for stage II and stage III colon cancer between October 2016 and October 2021 at Hospital Universitario La Paz (HULP), Madrid (Spain). The study protocol specified the inclusion criteria as follows: age above 18 years and completely resected colon adenocarcinoma located at >15 cm of the anal verge as determined by endoscopy or above the peritoneal reflection in the surgical resection without any evidence of metastatic disease. Main exclusion criteria were as follows: macroscopic evidence of residual tumor in the surgical specimen; no chemotherapy or radiotherapy were allowed before surgery; severe renal or hepatic disorder; bone marrow suppression; or disabling peripheral neuropathy. This study was approved by the Ethics Committee of HULP and was conducted in accordance with ethical standards of the Helsinki Declaration of the World Medical Association. Baseline disease, demographics, clinical data, treatment characteristics, and outcomes were analyzed from the medical record of each patient. Adjuvant chemotherapy was administered according to ESMO guidelines [4,9]. Patients were followed every 3 months with CT scan and CEA for the first 2 years from the surgery and every 6 months with CT scan and CEA from years 3 to 5. Colonoscopy was performed every 3 years starting 1 year after surgery.

The primary objective of the study was the identification of factors associated with time to recurrence (TTR). We chose TTR as the primary endpoint based on previous reports by other groups [8]. The sample was divided into a training cohort (patients diagnosed between October 2016 and September 2020, *n* = 440) and a validation cohort (patients diagnosed from October 2020 to September 2021, *n* = 100). TTR was calculated from the date of the surgery until the date of tumor recurrence or last follow-up. OS was defined as the time between the date of diagnosis and the date of death or last follow-up. The analysis was performed with a data cut-off of 15 September 2022. The relation between TTR and OS with each of the variables was analyzed using the log-rank test. Survival analysis was performed using the Kaplan–Meier method. Univariate cox regression analyses and multivariate proportional hazards regression model were carried out in the training cohort to identify independent prognostic factors for disease recurrence. We performed a correlation assessment using the Spearman’s rho test. Multicollinearity among variables was defined as a rho test value ≥ 0.50. In fact, we excluded adjuvant chemotherapy treatment as it positively correlates with the presence of high-risk features (Spearman’s rho test = 0.533; *p* < 0.001). In the multivariate analysis, we included the variables significantly associated with TTR in the univariate analysis. Multivariate analysis was performed with backward elimination. Prognostic factors that yielded a *p* value < 0.05 at the last step of multivariate cox regression analysis were included in the score. For the development of the score, each factor was assigned a particular score based on its β coefficient. The β coefficient for each risk factor was divided by the lowest β coefficient and rounded to the nearest whole number. Model calibration and discrimination were assessed in the training cohort by the area under the receiver operating characteristic (ROC) curve [10,11]. The final score of each patient was the sum of the points. The prognostic score was then applied to each patient. Survival by prognostic group was represented by Kaplan–Meier curves, and *p* values were calculated using the log-rank test. The training sample was divided into two risk strata (low-risk group and high-risk group) based on the approximate median of risk score. Hazard ratios (HRs) were calculated using cox proportional hazard regression, with *p* values calculated using the Wald statistics. The performance of the two-risk group strategy was tested for TTR in the validation cohort. All statistical analyses were carried out using SPSS v.25.

## 3. Results

A total of 440 patients with stage II and stage III colon cancer underwent curative surgery between October 2016 and October 2020 and were included in the training cohort. The baseline characteristics are depicted in Table 1. The median age at diagnosis was 74 years (range 35–95), and 44% of the patients were female. The primary tumor was distributed equally in the right and left colon. Stage II and stage III were observed in 50% percent of the patients each. Of note, preoperative CEA was available in 219 patients and was high in 19% of them. Twenty-five percent of the patients had high tumor budding. A total of 225 (51%) patients received adjuvant chemotherapy: 61 patients with stage II (27%) and 164 patients with stage III (75%).

After a median follow-up of 45 months (range, 0,1 to 66 months), 81 (18%) patients had a first tumor recurrence: 27 (12%) patients with stage II and 54 (24%) patients with stage III. Ninety-six (17%) patients died: 39 (17%) patients with stage II and 57 (26%) patients with stage III. The median TTR and OS were not reached for the whole cohort. Univariate cox regression analysis showed that T4 (tumor invades the visceral peritoneum or invades or adheres to the adjacent organ or structure), N2 (four or more regional nodes are positive) [12], R1 (incomplete tumor resection with microscopic surgical resection margin involvement) [13], bowel obstruction and perforation at diagnosis, lymphovascular and perineural invasion, high tumor budding (defined as ≥10 buds) [14], grade 3, and deficient mismatch repair were significantly associated with TTR. Only T4 (hazard ratio (HR), 3.46 [95% confidence interval (CI): 1.68 to 7.13], *p* < 0.01), N2 (HR, 2.29 (95%CI, 1.19 to 4.38), *p* = 0.01), and high tumor budding (HR, 1.91 (95%CI, 1.02 to 3.54), *p* = 0.04) remained with a *p* value <0.05 at the last step of the multivariate cox regression model, and were selected to create the clinical score (see Table 2).

**Figure 1:**
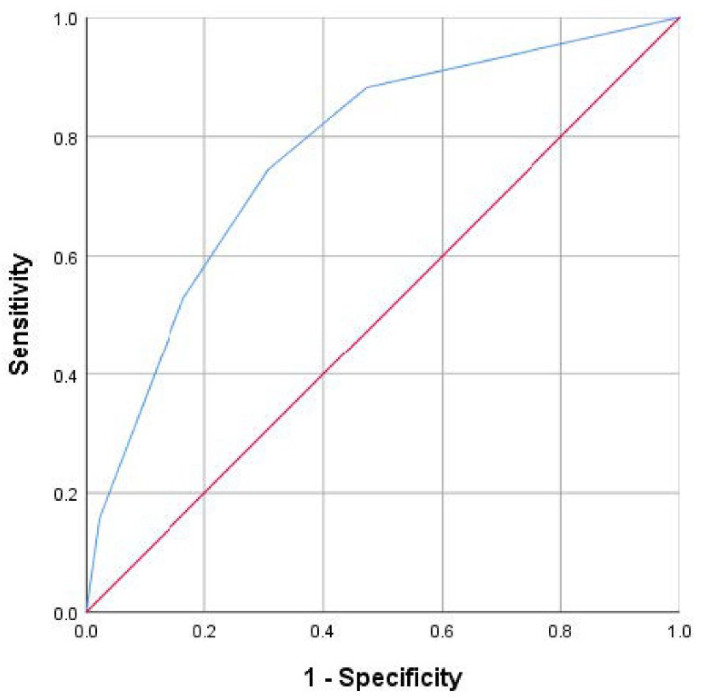
ROC curve of prognostic score (0 to 4 points) for recurrence at 24 months.

Based on the β coefficient of each feature (see Table 2), we assigned 2 points to T4, and 1 point to N2 and high tumor budding. Therefore, patients were assigned from 0 to 4 points (score 0 = 138, score 1 = 44, score 2 = 57, score 3 = 52, and score 4 = 13 patients). The area under the ROC curve for tumor recurrence at 36 months was 0.77 (95%CI, 0.70 to 0.84), *p* < 0.01 (Figure 1).

**Figure 2:**
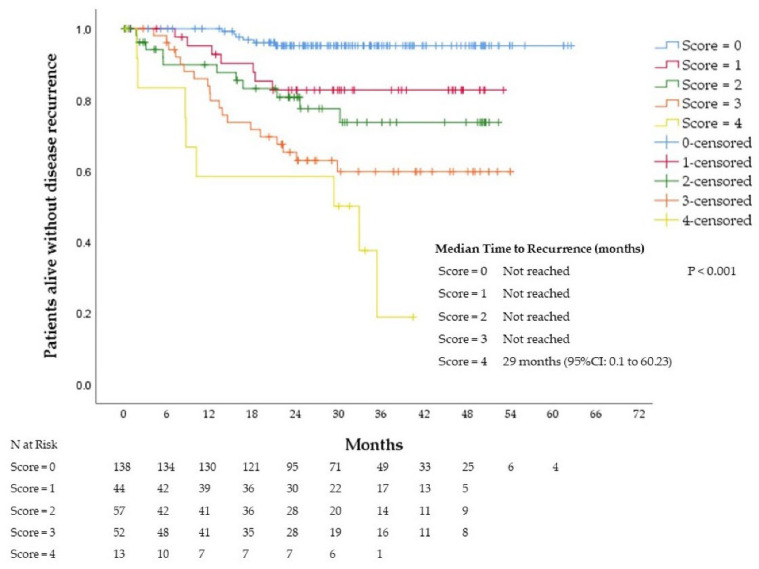
Time to recurrence according to the score (0–4) in the training cohort.

**Figure 3:**
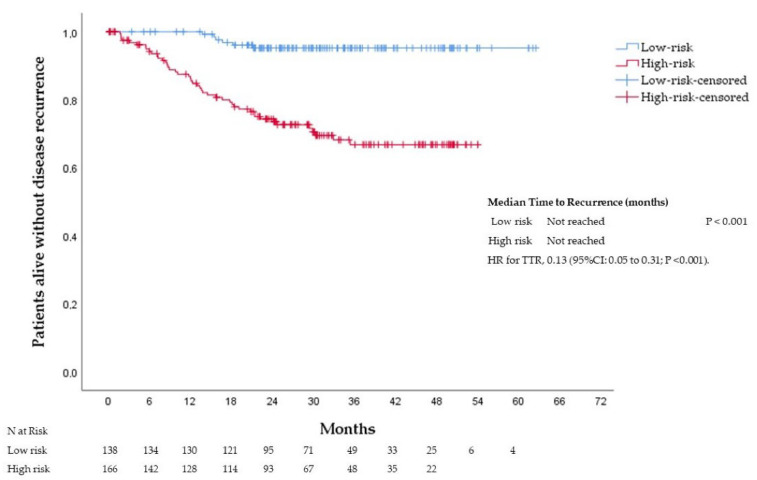
Time to recurrence according to risk groups (low-risk vs. high-risk) in the training cohort. HR, hazard ratio; CI, confidence interval.

At 36 months, 95%, 83%, 73%, 60%, and 19% of the patients with scores 0, 1, 2, 3, and 4 were recurrence-free, respectively. The median TTR was not reached in patients with scores 0−3. Patients with score 4 had a median TTR of 29 months (95% confidence interval (CI): 0.1 to 60.23). Significant differences were observed between the groups (*p* < 0.001), see Figure 2. Patients were divided into a low-risk group (score = 0; *n* = 138; 45% of the patients) and a high-risk group (score = 1−4; *n* = 166; 55% of the patients). At 36 months, 95% and 67% of the patients in the low-risk and high-risk groups were recurrence-free, respectively. Patients assigned to the low-risk group had a significantly longer TTR than patients assigned to the high-risk group. The median TTR was not reached in either group, with a HR for disease recurrence of 0.13 (95%CI: 0.05 to 0.31; *p* <0.001), see Figure 3.

**Figure 4:**
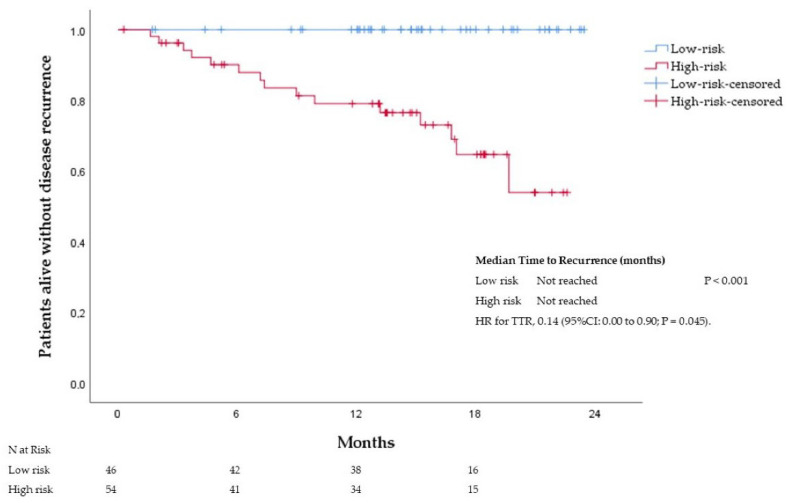
Time to recurrence according to risk groups in the validation cohort.

A total of 100 patients were included in the validation cohort. The baseline characteristics are depicted in Table 1. The median age at diagnosis was 75 years (range 45–97), and 50% of the patients were female. The primary tumor was distributed equally in the right and left colon. Stage II was observed in 54% percent of the patients each. Twenty-one percent of the patients had high tumor budding. A total of 43 patients received adjuvant chemotherapy. Patients were assigned to the low-risk (*n* = 46; 46%) and high-risk (*n* = 54; 54%) groups. After a median follow-up of 15 months (range, 2 to 25 months), 15 (15%) of the patients had a first tumor recurrence. Recurrences were observed in five (9%) patients with stage II and 10 (22%) patients with stage III. According to our score, all the recurrences were observed in the high-risk group. At 12 months, 100% and 79% of the patients in the low-risk and high-risk groups were recurrence-free, respectively. Patients assigned to the low-risk group had significantly longer TTR than patients assigned to the high-risk group. The median TTR was not reached in either group. HR for disease recurrence of 0.14 (95%CI: 0.00 to 0.90; *p* <0.045), see Figure 4.

## 4. Discussion

In this study, we created a simple score using three clinicopathological parameters available in routine clinical practice to better estimate the recurrence risk in patients with stage II and stage III colon cancer. This score shows that the probability of recurrence ranges from 5% in patients with a score = 0 to 81% in patients with a score = 4, with an AUC of 0.77. More importantly, the score can discriminate a subgroup of patients (low-risk group, score = 0), so that even with locally advanced disease, they will have an excellent prognosis after completing the standard treatment recommendations according to their stage. This low-risk group comprises the 45% of the training cohort included in the multivariate analysis and 46% of the patients in the validation cohorts.

Multiple scores and nomograms have attempted to overcome the aforementioned limitations of the AJCC’s TNM staging system for the prediction of outcomes. One of the most relevant is the MSKCC nomogram published in 2008 for the estimation of the recurrence risk of patients with stages I to III colon cancer after a complete resection (R0) of the tumor [6]. The nomogram was based on nine variables including patient age, tumor location, preoperative carcinoembryonic antigen, T stage, number of positive and negative lymph nodes, lymphovascular invasion, perineural invasion, and use of postoperative chemotherapy. The nomogram successfully predicted relapse with a concordance index of 0.77, improving the stratification provided by the AJCC staging scheme and was externally validated in multiple cohorts [15,16,17]. However, the high number of features and its complexity may prevent it from being used as a practical tool in clinical practice. The MSKCC clinical calculator was updated in 2019 [8]. The nomogram was simplified to six variables and incorporated recently validated molecular and histologic factors, including microsatellite genomic phenotype; AJCC T category; number of tumors involved; lymph nodes; presence of high-risk pathologic features, such as venous, lymphatic, or perineural invasion; presence of tumor-infiltrating lymphocytes; and use of adjuvant chemotherapy. The concordance index was 0.792, and external validation confirmed the utility for the prediction of recurrence. Unfortunately, the generalization of this nomogram was hampered because tumor-infiltrating lymphocytes are not reflexively measured in many centers, including ours.

Our score was built with variables that showed a *p* value < 0.05 in the multivariate cox regression model and included T4, N2, and budding. Primary colon cancer is classified as T4 per the AJCC TNM staging 8th edition when it invades the visceral peritoneum or invades or adheres to an adjacent organ or structure [12]. T4 has classically been considered a negative prognostic factor. In fact, patients with T4 stage II disease have worse outcomes than patients with stage IIIa disease. A recent subanalysis of patients with stage II colon cancer included in the IDEA collaboration showed that high-risk stage II patients with T4 disease have a worse outcome than those with T3 disease [18]. The IDEA collaboration also highlighted that those patients with stage III with T4 and/or N2 are a different population with a worse prognosis than the other patients with stage III (T1−3 and N1) and suggested the use of these risk groups as stratification categories in randomized trials [19]. Tumor budding refers to isolated or clusters of up to four cancer cells located at the invasive tumor front [20]. A growing amount of evidence has confirmed its prognostic value in localized colon cancer, independent of the tumor grade [21,22]. A recently published subanalysis from the IDEA-France phase III trial [23] showed that tumor budding is an independent prognostic factor in stage III colon cancer patients. The DFS at 3 years was 79% vs 67% (*p* = 0.001) in patients with budding grade 1 vs 2−3 with a HR for recurrence or death of 1.41 (95% CI, 1.12 to 1.77), *p* = 0.003, after adjustment for relevant clinicopathological features. Interestingly, high tumor budding was associated with perineural (*p* < 0.01) and vascular (*p* = 0.002) invasions, which may explain that these well-known adverse prognostic features are not present in the last step of our multivariate analysis. The role of tumor budding in predicting benefit from adjuvant chemotherapy is still controversial. In a subanalysis of the SACURA trial [24], a nonsignificant improvement of 5% in the 5-year recurrence rate was observed in patients with stage II and stage III colon cancer treated with adjuvant chemotherapy vs surgery alone. In patients with pT1, tumor budding currently influences decision making. More recently, the ASCO guidelines were updated and added high tumor budding (≥10 buds, high grade) to the list of adverse prognostic factors to classify patients in the high-risk subpopulation that may derive more benefit from chemotherapy [25]. However, the ESMO guidelines for the management of localized stage II colon cancer still do not consider tumor budding in the decision making. In light of the results of our group and those of previous groups, high tumor budding might be considered as a risk factor.

Prognostic characterization and subgroup categorization in patients with localized colon cancer have more implications than providing the patient a tailored risk of recurrence. Some authors suggest that stratification categories based on T and N should be included in randomized trials for localized colon cancer. Assessing the risk of recurrence may also have implications for the follow-up. The ESMO guidelines recommend a CT scan of the chest and abdomen every 6 to 12 months for the first 3 years in patients who are at higher risk of recurrence according to the TNM classification. Other authors suggest that the preferred approach should be performing two CT scans at 12 and 36 months independent of the stage and risk groups due to the lack of survival benefit of a more intensive approach [26]. We suggest that due to the significantly different risk of recurrence according to subgroups, and the possible benefit of early treatment of oligometastatic disease, the follow-up should be tailored accordingly, or at least taken into account in future follow-up trials.

The limitations to our study are mostly due to its retrospective and unicentric nature. A significant amount of data are missing including tumor budding and preoperative CEA, mainly in the training cohort. Preoperative CEA should be performed before surgery; however, data are missing in half of the patients due to multiple reasons including emergency surgery or even human error. The advantage of our score is that it is based on three features that should be available in every patient with a colorectal cancer diagnosis. Adjuvant treatment was given to the patients following indications by the ESMO guidelines [4,9]. This feature was not considered in the analysis because the benefit of therapy may be masked by the administration in the high-risk subgroup. In fact, we found a correlation between the presence of high-risk features as defined by the ESMO guidelines and the administration of adjuvant chemotherapy (Spearman’s rho test = 0.533; *p* < 0.001). Therefore, this score should not be interpreted as a predictive marker of benefit for adjuvant chemotherapy but rather as a predictive marker for recurrence in patients that have followed the standard treatment strategy for localized colon cancer. Nevertheless, we consider that tumor budding is such a strong predictive marker for recurrence that should also be considered as a risk factor and should be included in the guidelines for adjuvant chemotherapy. Only patients with complete (R0) resection were included in the initial MSKCC nomogram [6]; however, approximately 10% of patients have involved resection margins at the pathological report of the surgery. We therefore consider that this feature should be included in a real-world analysis. Finally, although the results of the internal validation cohort seem to confirm the performance of our score, the sample size was small, and the cohort was still immature. We did not perform an external validation and thus an accurate determination of the AUC and calibration of the model was not possible.

## 5. Conclusions

In conclusion, defining subgroups of patients with localized colon cancer at a high risk of recurrence has implications in the treatment strategy, trial designs, and follow-up. Although the traditional AJCC TNM staging provides adequate prognostic estimation, a more personalized approach using high-risk clinicopathological features may be more precise and practical. In our study, we built a simple score to accurately predict tumor recurrence based on T4, N2, and high tumor budding. Patients with a score = 0, that comprises 44% of the cohort, had an excellent prognosis. A longer follow-up is needed, and an external validation is recommended to confirm our results.

## Figures and Tables

**Table 1:** Baseline characteristics of the patients.

Characteristic (*n* = Training Cohort)	Training Cohort (*n* = 440)	Validation Cohort (*n* = 100)
Sex (female)	193 (44)	50 (50)
Age Age < 50	74 (35–95) 22 (5)	75 (45–97) 4 (4)
Location Right Left	212 (48) 228 (52)	56 (56) 44 (44)
Stage at diagnosis II III	222 (50) 218 (50)	54 (54) 46 (46)
T 1 2 3 4	5 (1) 17 (4) 252 (58) 163 (37)	1 (1) 3 (3) 50 (50) 46 (46)
N 0 1 2	222 (50) 150 (34) 68 (16)	58 (58) 33 (33) 9 (9)
R0	413 (94)	93 (93)
Preoperative CEA >5 ng/ml	41 (19)	10 (20)
Bowel obstruction at diagnosis	45 (10)	15 (15)
Bowel perforation at diagnosis	37 (8)	5 (5)
Lymphovascular invasion	184 (43)	47 (47)
Perineural invasion	85 (20)	31 (31)
Budding Low Medium High	142 (47) 84 (28) 78 (25)	49 (49) 30 (30) 21 (21)
Grade 1 2 3	18 (4) 367 (88) 33 (8)	1 (1) 91 (91) 8 (8)
Mucinous	82 (16)	19 (19)
Rignet cell	13 (3)	5(5)
≥12 resected lymph nodes	382 (90)	96 (96)
dMMR	68 (17)	14 (14)

CEA, carcinoembryonic antigen; dMMR, deficient mismatch repair; R0, complete tumor resection with all margins histologically uninvolved.

**Table 2:** Multivariate Cox Regression Analysis.

Characteristic	β Coefficient	HR (95%CI)	*p* Value
T4	1.243	3.46 (1.68–7.13)	0.001
N2	0.829	2.29 (1.19–4.38)	0.012
High tumor budding	0.647	1.91 (1.02–3.54)	0.041

## Data Availability

The data presented in this study are available on request from the corresponding author.
